# Heavy-atom labeling of RNA by PLOR for *de novo* crystallographic phasing

**DOI:** 10.1371/journal.pone.0215555

**Published:** 2019-04-15

**Authors:** Jason R. Stagno, Ping Yu, Marzena A. Dyba, Yun-Xing Wang, Yu Liu

**Affiliations:** 1 Protein-Nucleic Acid Interaction Section, Structural Biophysics Laboratory, Center for Cancer Research, National Cancer Institute, National Institutes of Health, Frederick, Maryland, United States of America; 2 Basic Science Program, Leidos Biomedical Research, Inc., Frederick National Laboratory for Cancer Research, Frederick, Maryland, United States of America; 3 State Key Laboratory of Microbial Metabolism, School of Life Sciences and Biotechnology, Shanghai Jiao Tong University, Shanghai, People’s Republic of China; Universitetet i Bergen, NORWAY

## Abstract

Due to the paucity of known RNA structures, experimental phasing is crucial for obtaining three-dimensional structures of RNAs by X-ray crystallography. Covalent attachment of heavy atoms to RNAs is one of the most useful strategies to facilitate phase determination. However, this approach is limited by the inefficiency or inability to synthesize large RNAs (>60 nucleotides) site-specifically labeled with heavy atoms using traditional methods. Here, we applied our recently reported method, PLOR (position-selective labeling of RNA) to incorporate 5-iodouridine at specific positions in the adenine riboswitch RNA aptamer domain, which was then used for crystallization and subsequent *de novo* SAD phasing. PLOR is a powerful tool to improve the efficiency of obtaining RNA structures *de novo* by X-ray crystallography.

## Introduction

The ongoing discovery of numerous biologically important RNAs demands the determination of their structures to better understand their functions. X-ray crystallography contributes the majority of RNA three-dimensional structures in the PDB [1]. Solving the phase problem is the major bottleneck between X-ray diffraction data and refined structures. Obtaining phase information in the case of RNA is often challenging, and most often requires *de novo* phase determination using anomalous or isomorphous difference data from heavy-atom derivatives [2–5]. Heavy-atom derivatization strategies have been widely used to enable crystallographic phasing [1, 2, 6]. Soaking RNA crystals in buffers containing heavy atoms (e.g., halides, metal ions) offers a straightforward approach to introduce anomalous scatterers into the ordered solvent shell surrounding the RNA molecules. However, not all crystals can tolerate such soaking, which may result in crystal cracking, deterioration, and loss in diffraction quality. In addition, heavy-atom incorporation by soaking is not guaranteed, and the number and location of binding sites is often unpredictable. Engineered sequences, for example, a tandem G-U wobble pair cation binding motif, can be inserted into RNAs to improve the binding specificity of heavy-atoms [2]. Such strategies require mutations in some non-functional regions of an RNA molecule. Alternatively, RNA molecules can be derivatized with halogenated nucleotides by incorporating them during RNA synthesis. Modified RNAs are usually prepared by *in vitro* transcription or solid-phase chemical synthesis methods. *In vitro* transcription can produce RNAs with lengths varying from tens to thousands of nucleotides (nt), but it is usually applied for producing RNAs labeled specifically by nucleotide type rather than at selected positions. Solid-phase chemical synthesis is the most widely used method for preparing site-specifically labeled RNAs. However, heavy-atom labeled phosphoramidites are not always commercially available, and it is challenging to incorporate with adequate efficiency heavy atoms at specific positions of RNAs larger than 60 nt in length using step-wise chemical synthesis [7]. This size limitation can be alleviated by enzymatically ligating short synthetic RNAs fragments [8]. However, discrete and complicated labeling schemes may be difficult to achieve by ligation due to low efficiencies and limited site choices.

To overcome limitations in phasing RNA structures *de novo* by X-ray crystallography, we applied the position-specific labeling of RNA (PLOR) method to incorporate heavy atoms at desired positions in RNA. PLOR is a solid-liquid hybrid transcription method for preparing position-specifically labeled RNAs [9–11]. All chemicals used in PLOR are commercially available, which obviates the need for labor-intensive organic synthesis. In principle, PLOR can be used to site-specifically label RNAs with no size limitations, as with conventional *in vitro* transcription; it also allows for incorporation of functional groups, including bulky ones such as fluorophores, into RNAs as long as they can be tolerated by RNA polymerase. Due to the high costs of synthesizing heavy-atom labeled RNAs, initial crystallization trials are typically performed using their unmodified counterparts. However, in many cases, introducing heavy-atoms can alter crystallization conditions, thus requiring additional screening with the modified RNA sample. The capability of PLOR to generate large amounts of site-specifically labeled RNAs at low cost and high efficiency saves time, effort, and materials required for such crystallographic studies. We reported a general strategy for heavy-atom derivatization of RNA using PLOR to selectively incorporate 5-iodouridine (IU) at two positions in the adenine riboswitch aptamer domain (rA71) [10]. Here we demonstrate that incorporation of a small number of halogen atoms generates enough anomalous phasing power for *de novo* structure determination, while minimizing RNA structural perturbations and effects on crystal quality.

## Results

### Selective incorporation of halogenated nucleotides into RNA

**Fig 1:**
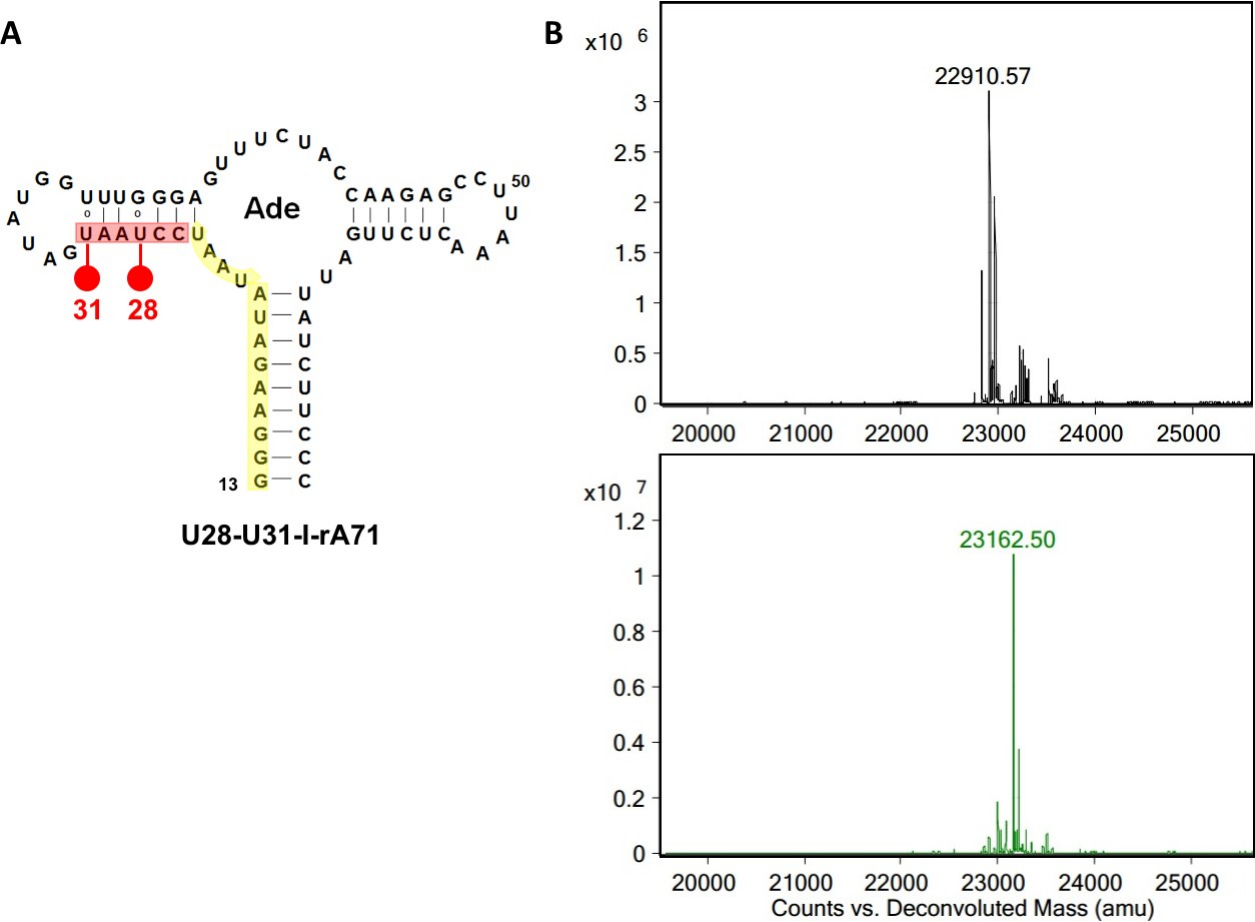
Incorporation of 5-iodouridine into rA71. (A) Secondary structure of U28U31-I-rA71. The iodine atoms are represented by red dots at positions 28 and 31. Residues highlighted in yellow represent the first 13 nucleotides synthesized in the initiation stage. (B) The ESI-Mass spectra of non-labelled rA71 (top) and the U28U31-I-rA71 (bottom).

PLOR overcomes the drawbacks of other existing synthetic techniques, and can be applied as a routine strategy for preparing heavy-atom derivatives of RNA with readily available reagents and simple sample purification. Incorporation of halogenated pyrimidines was our first application of PLOR for crystallographic phasing studies. In the PLOR synthesis of U28-U31-I-rA71, micromolar concentrations of NTPs were used in the elongation (10 μM ATP/CTP/I-UTP) and termination (100~200 μM NTPs) stages, which are much lower than the millimolar concentrations used routinely in conventional transcription. Such low usage of NTPs, especially expensive modified NTPs such as I-UTP, greatly reduces the costs of synthesizing selectively labeled RNAs. The overall yield for PLOR-generated U28-U31-I-riboA71 was approximately 37.5%. The expected yield for this synthesis is 35.5% (calculated as Yield = *I*×*E*^*n*^, where *I* and *E* are the percent efficiencies of initiation and elongation, respectively, and *n* is the total number of cycles used for synthesis [9]). This suggests that T7 RNAP can tolerate I-UTP equally as well as UTP. Deconvoluted ESI-Mass spectra of U28-U31-I-rA71 and its native counterpart, rA71 (generated by conventional *in vitro* transcription) showed major m/z peaks of 23,162.50 Da (Fig 1B, bottom), and 22,910.57 Da (Fig 1B, top), respectively, whose difference (251.93 Da) matches that of the mass difference between two iodines and two hydrogen atoms (251.8 Da). In addition, U28-U31-I-rA71 and rA71 exhibited indistinguishable migration by native PAGE (not shown), suggesting that the incorporation of IU at the two positions had little impact on RNA folding.

### Structure solution and refinement

**Fig 2:**
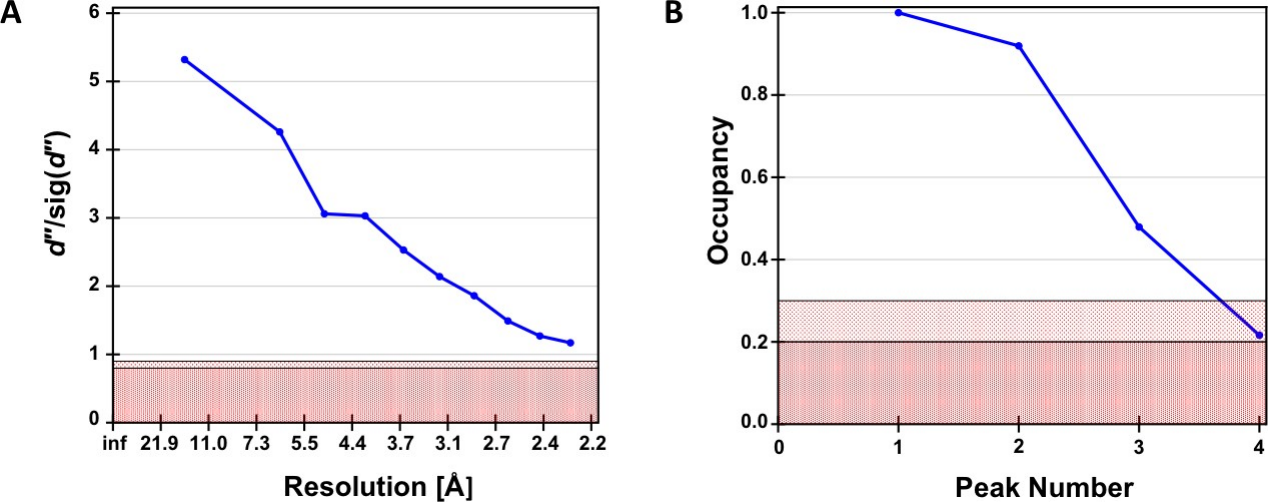
*HKL2MAP* profiles obtained from *SHELXC* and *SHELXD*. *HKL2MAP* profiles obtained from *SHELXC* and *SHELXD*. (A) *dʺ*/sig(*dʺ*) as a function of resolution from *SHELXC*. (B) Site occupancies of peaks found by *SHELXD*. Of the four sites identified, the two correct sites, peaks 1 and 2, correspond to I-U28 and I-U31, respectively, each with a relative occupancy of >90%.

**Fig 3:**
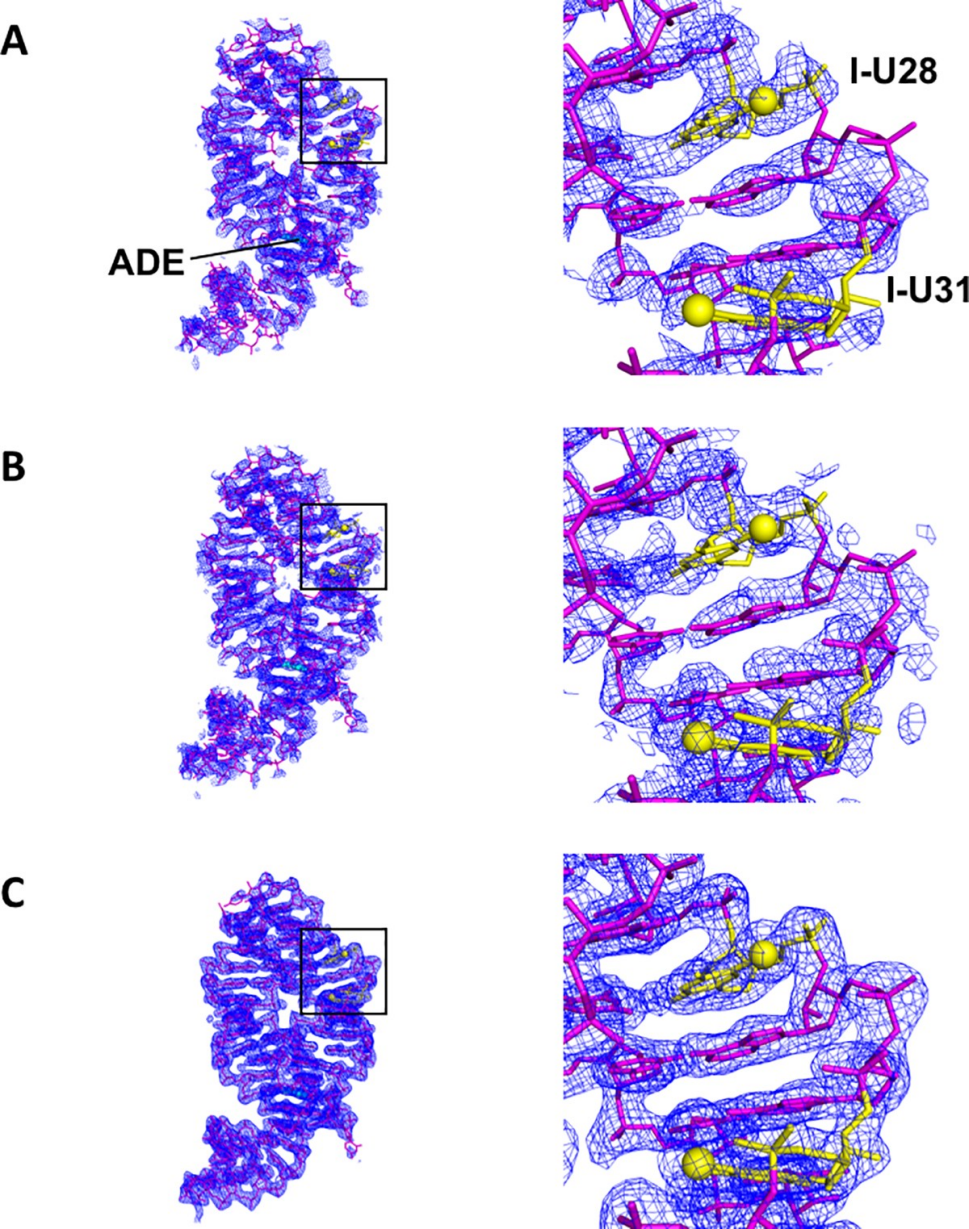
SAD *de novo* phasing and improvements in electron density. Electron-density map 2F_o_-F_c_, 1.0 σ contour) computed using the phases from (A) *SHELXE*, (B) *PHENIX autosol*, and (C) final refinement in *PHENIX refine*. The initial maps from phasing, without density modification, were sufficient for automated model building using *PHENIX autobuild*. The refined model of U28U31-I-rA71 is shown as a stick model (magenta) with adenine ligand as cyan spheres. Labeled positions are depicted in yellow with the iodine atoms displayed as spheres.

Crystal data were reduced to 2.22 Å using *HKL2000* [12] in the orthorhombic space group, *P*2_1_2_1_2, with unit-cell dimensions very similar to the non-labeled rA71 crystals (PDB code: 4TZX [13]). *PHENIX xtriage* [14] was used to assess data quality, with anomalous measurability up to ~2.4 Å. The heavy-atom substructure was determined by single anomalous dispersion (SAD) using *SHELXC/D/E* [15] through the graphical interface, *HKL2MAP* [16]. *SHELXC* estimated the detectable anomalous signal (*dʺ*/sig(*dʺ*) >1.8) to ~2.7 Å (Fig 2A). The top substructure solution (CFOM: 43.81) from *SHELXD* consisted of four heavy atoms, two of which had occupancies > 0.9 (Fig 2B), and initial phases were generated using *SHELXE* (Fig 3A). The phases were improved by SAD phasing with *PHENIX AutoSol* [14] using the top two heavy atoms (occupancy >0.9) from *SHELXD* (Fig 3B). With the improved phases, *PHENIX autobuild* [14] successfully built 50 out of the 71 nucleotides. The remainder of the structure was built manually using COOT [17] with iterative cycles of refinement in *PHENIX refine* [14], which included TLS parameters and the refinement of heavy-atom occupancies and anomalous scattering factors. Crystal data and atomic coordinates were deposited in the PDB under accession code 5UZA.

**Fig 4:**
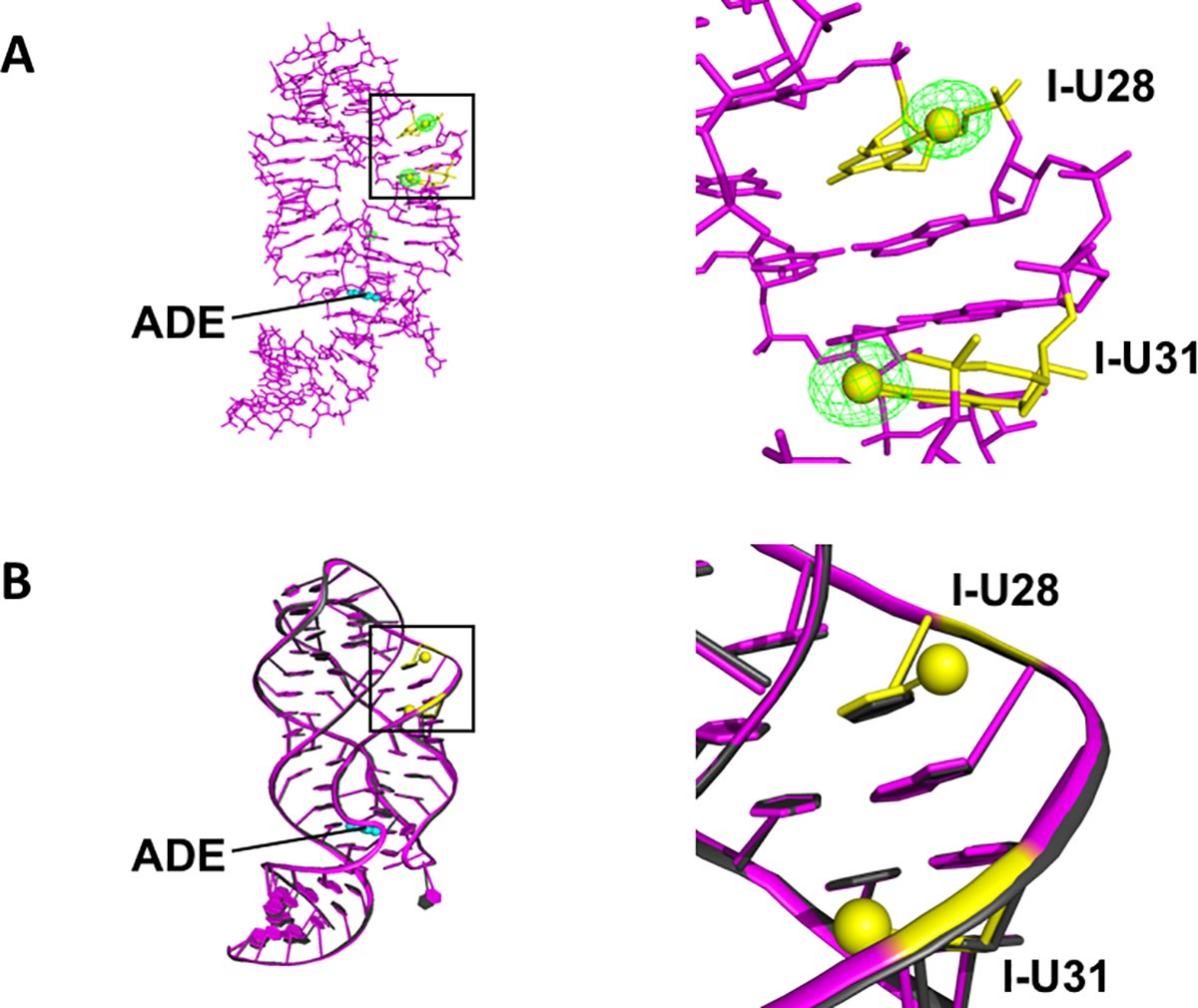
Structural analysis and comparison. (A) Anomalous difference Fourier map (green), contoured at 5 σ, calculated from *ANODE*. The refined model of U28U31-I-rA71 is shown as a stick model (magenta) with adenine ligand in cyan spheres. Labeled positions are depicted in yellow with the iodine atoms displayed as spheres. (B) Superposition of U28U31-I-rA71 (magenta) and non-labelled rA71 (black, PDB: 4TZX), shown in cartoon. Incorporation of 5-iodourdine at the two positions causes no structural perturbations nor any significant effects on crystal quality.

Crystals of U28-U31-I-rA71 proved resilient to radiation damage after an average dose of photons for a routine SAD dataset. This is particularly important since a high redundancy of measurements is often required for anomalous phasing of crystallographic data, which can be problematic for heavy-atom derivatives that are more susceptible to radiation damage. However, with only two iodines per ~23 kDa RNA molecule, and an 8.5-fold overall data redundancy, the anomalous signal was sufficiently measurable and complete for almost the entire resolution range. The anomalous difference Fourier (Fig 4A), generated using *ANODE* [18] revealed peak heights of 24 and 19 σ for I-U28 and I-U31, respectively. Comparing the structures of U28-U31-I-rA71 and non-labeled rA71(PDB code: 4TZX) [13] demonstrates that no local structural perturbations are induced by the heavy-atom modifications (Fig 4B). The resolution of U28-U31-I-rA71 (2.22 Å) is comparable to that of the unmodified RNA (2.01 Å), as is the data quality: *R*_work_/*R*_free_ = 0.215/0.251 (labeled) compared to 0.224/0.257 (non-labeled). Importantly, this indicates that the introduction of iodines at the two positions has little, if any, effect on crystal quality.

For comparison, experimental phasing was also attempted using the single isomorphous replacement with anomalous scattering (SIRAS) method. The unit cell dimensions for the derivative crystal are *a* = 48.99, *b* = 151.58, *c* = 24.74, corresponding to fractional differences relative to the native crystal [13] of 1.3%, 0.8%, and 1.0%, respectively, and a mean fractional isomorphous difference in intensities (*R*_int_) [15] of 46.6%. Despite the poor isomorphism, *SHELXD* using the SIRAS method correctly identified the heavy-atom sites, but only when the resolution was limited to 3.5 Å. However, phasing in *SHELXE* using the correctly identified sites and phase extension to 2.01 Å with the native data failed to generate an interpretable electron density map. In this case, therefore, non-isomorphism precluded *de novo* phasing by SIRAS.

## Discussion

The process for obtaining heavy-atom modified RNAs is a key bottleneck in solving unique biologically important RNA structures by X-ray crystallography. The introduction of heavy-atoms into RNA can be routinely achieved by PLOR, with the aim of breaking the phase ambiguity of crystallographic data. Such an application for PLOR is especially feasible for large RNAs that cannot be synthesized by solid-phase synthesis with high-enough efficiency. The easy scale-up of PLOR also makes it practical to carry out initial crystallization screening with heavy-atom containing RNAs. This avoids the potential pitfalls of non-isomorphism between native and derivative crystals. Covalent incorporation of heavy atoms into RNA at desired positions using PLOR offers a general strategy for SAD/MAD phasing of novel RNA crystal structures. This application of PLOR can be extended to structure determination of RNA/protein complexes by derivatizing RNA instead of, or in addition to, the protein, thereby increasing the chances for successful *de novo* phasing. It is straightforward to extend the strategy to synthesize RNAs containing other heavy-atoms, including Cl, Br, Se, *etc*. by PLOR. This application of PLOR will hasten the rate of novel RNA structure determination by X-ray crystallography.

## Materials and methods

### Sample preparation

A previous study showed that phase information could be obtained for a crystal of a 160-nt RNA using the anomalous signal from four iodines [19]. To minimize potential structural perturbations, we chose to label rA71 (71nt) with IU at two positions, 28 and 31, which reside in the second duplex (highlighted in red, Fig 1A). The procedure for the preparation of labeled RNA (U28-U31-I-rA71) by PLOR was as described previously [10]: a 5 mL PLOR reaction, consisting of one initiation, one elongation, and one termination cycle. In the initiation cycle, 10 μM T7 RNA Polymerase (T7 RNAP), 10 μM solid-phase rA71 DNA template (biotinylated DNA immobilized on neutravidin-coated agarose beads), 0.96 mM ATP, 0.96 mM GTP and 96 μM UTP were incubated at 37°C for 15 min to initiate synthesis, producing the first 13 nucleotides (highlighted in yellow in Fig 1A). Filtration and bead-rinsing were performed multiple times to remove the residual NTPs after the initiation cycle. In the elongation (labeling) cycle, 20 μM ATP, 20 μM CTP and 20 μM 5-iodouridine triphosphate (TriLink BioTechnologies) were added to the solid-phase transcription complexes generated in the initiation cycle, and the reaction was incubated for 10 min at 25°C, extending the transcripts to I-U31 (highlighted in red in Fig 1A), followed by filtration and bead-rinsing. In the termination cycle, 120 μM ATP, 110 μM CTP, 100 μM GTP and 170 μM UTP were mixed with the complexes at 25°C for 12 min to complete the transcription of U28-U31-I-rA71. ~0.30 mg of labeled RNA was obtained after purification by phenol/chloroform extraction, and was exchanged into buffer (pH 6.8) containing 50 mM KOAc, 100 mM MgCl_2_, 1 mM spermine and 5 mM adenine. The incorporation of the iodine atoms was verified by mass spectrometry (Fig 1B).

### Crystallization and data collection

Crystals of U28-U31-I-rA71 were grown at 4°C by sitting-drop vapor diffusion using as the reservoir 50 mM Tris-HCl (pH:8.5), 100 mM KCl, 10 mM MgCl_2_ and 30% (v/v) Polyethylene Glycol 400 (PEG 400). The RNA stock concentration used for crystallization was 0.5 mM and the volume ratio between RNA and reservoir for the sitting drops was 1:2. U28-U31-I-rA71 crystals appeared as rectangular plates in ~2–3 days, and grew to maximum dimensions of ~100×25×15 μm^3^. Crystals for data collection were flash-frozen directly in liquid nitrogen, without additional cryoprotection. Diffraction data were collected at beamline 19BM, Advanced Photon Source (APS), Argonne National Laboratory, using a photon energy of 8 keV (1.54 Å) and an exposure time of 1 s per 0.5°, for a total of 240° (480 frames). The crystals diffracted to a maximum resolution of ~2 Å and exhibited no signs of radiation damage. Crystallographic data and refinement statistics are summarized in Table 1. Structure figures were generated with *PyMOL* [20].

**Table 1:** Data collection and refinement statistics.

	U28-U31-I-riboA71 (PDB: 5UZA)
**Data collection**	
Beamline	19BM, APS
Space group	*P*2_1_2_1_2
Unit-cell parameters (Å, ^o^)	a = 49.0, b = 151.6, c = 24.7, α = β = ɣ = 90
Wavelength (Å)	1.54
Resolution (Å)	50.00–2.22 (2.30–2.22)
No. of unique reflections	9770 (932)
Completeness (%)	99.7 (99.3)
Multiplicity	8.5 (5.2)
*I*/σ(*I*)	43.3 (2.1)
*R*_merge_ (%)	4.8 (65.8)
*R*_p.i.m._ (%)	1.7 (30.7)
*CC*_1/2_ (outer shell)	0.78
**Refinement**	
Resolution (Å)	46.62–2.22
*R*_work_/*R*_free_ (%)	21.5/25.1
R.m.s.d. bond lengths (Å)	0.005
R.m.s.d. bond angles (^o^)	0.386
Mean *B* factor (Å^2^)	42.0
Wilson *B* factor (Å^2^)	37.0
No. of atoms (non-H)	1573
RNA	1505
Magnesium	3
Water	55
Ligand	10

Values in parentheses are for the highest resolution shell.
